# Supplementary material for: Patient and Therapist Perspectives on Treatment for Adults with PTSD from Childhood Trauma
Source: J Clin Med. 2021 Mar 1;10(5):954. doi: 10.3390/jcm10050954 (PMC7957589; doi:10.3390/jcm10050954)
Supplement: Supplementary file 1 [file jcm-10-00954-s001.pdf]

Journal of
Clinical Medicine

MDPI

**Supplement 1: Patient Interview Questions**

The questions I will ask are to try and explore what might have changed for you and how this relates to your treatment. Just a reminder there are no right or wrong answers, I am just interested in your thoughts on the treatment experience and the impact this has had on your life.

**Change experienced**

1. If you think back to before you started treatment, how are things going?
   Prompts 1a. How are you doing now in compared to when you started?
2. What changes have you noticed in your life since having treatment?
   2a. What about things like your mood? Feelings? Thoughts? Behaviours?
   2b. What about how you view or think about yourself?
   2c. What about your relationships?
   2d. Is there any change to your outlook on life?
   2e. Do you think about your trauma experiences different? i.e. have a different perspective, how you feel…..?

**Nature of change**

3. You mentioned that you have experienced changes in your life as a result of your therapy. Can you tell me about how these changes occurred?
   Prompts 3a. Was there a particular point where things changed? Or were the changes more gradual?
   3b. What was the shift about? What happened or changed?
   3c. What else was happening around then?
   3d. Were there any changes to the connections you had either in therapy or outside?
   3e. Have all the changes remained?
   3f. Do you think that these changes will be lasting?
   Why/ why not?

**Now I would like to ask you some questions about your treatment**

*Preparation*

4. What are your thoughts on your introduction to the treatment you were going to receive?
   Prompts 4a. Did you feel you were prepared/ready to start treatment?
   4b. Did you feel safe/supported enough to explore trauma experiences?

*Therapy*

5. Can you tell me about the therapy you received and your thoughts about it?
   Prompts 5a. What was helpful about therapy?
   5b. What was unhelpful about therapy?
   5c. How do you think therapy helped you?
   5d. Did it have any effect on the issues you wanted to address?
   5e. Did you get what you needed from therapy?
   Tell me more about that?
   5f. Was there anything that you feel could have been done differently? Maybe something added or left out?
   5g. Would you recommend this treatment to someone else?

*Difficulty during therapy*

6. In terms of your experiences during therapy, were there any challenges, which made it difficult for you?
   Prompts 6a. What kind of problems did you have?
   6b. How did you manage them?

6c. Were these problems related to addressing your childhood experiences? Or were there other things going on in your life outside of therapy?

6d. If things got hard or started to become too much, what made you continue?

*Therapist*

7. What are your thoughts about your therapist?

Prompts 7a. What did they do that was helpful?

7b. What did they do that was unhelpful?

7c. If, things became difficult how did they manage this or help you?

**I want to ask a bit about your reasons for coming to get help related to your childhood trauma experiences**

*Expectations and motivation*

8. What made you come to therapy when you did?

8a. Prior to treatment, did you think you could change?

*Psychological treatment experiences*

9. Have you ever been to see someone about your trauma history before?

9a. Have you ever had therapy that specifically focused on addressing your trauma experiences?

If yes, how did the therapy that you received in the study compare to other therapy or counselling that you have received?"

9b. Do you think you will have more psychotherapy because of your trauma experiences?

If yes, what aspects of this do you feel you still need to work on?

Can you tell me why you think that this wasn't addressed during your treatment?

9c. What would you have done differently given the chance?

**I want to ask a few questions about the treatment approach**

*Trauma-focused treatment*

The treatment you received is considered a 'trauma-focused' treatment. This means that the treatment is focused on directly reprocessing people's trauma memories and experiences.

10. What was your experience of this?

Prompts 10a. Did you think this approach was appropriate? i.e. did you find it difficult?

10b. If you had to do it again would you be happy to receive the same kind of treatment?

Some people think that trauma-focused treatment is not appropriate for people who have experienced childhood trauma.

11. What are your thoughts on this view?

Prompts 15a. Do you agree with this?

Why/ why not?

15b. Do you think this approach would be appropriate for everyone?

Why/ why not?

**Any other comments**

This brings us to the end of the interview. Do you have any other comments you would like to make in regards to your experiences?

**Supplement 2: Therapist Interview Questions**

**Overall experience**

**First I would like to ask you a few questions in relation to your background and experience**

1. What is your experience of treating Complex PTSD?
2. Overall can you describe what has been your experience of conducting EMDR/ImRs therapy? Any experience with other Tf-approaches?

**Change**

**These next few questions are about the overall nature of change and what factors are important to this process – not necessarily treatment specific factors**

3. What do you think are the important components of therapy that facilitate change?
4. What role do you as a therapist take to help this process?

**Treatment**

**Now I would like to ask you some questions that are specific to the treatments**

5. Can you tell me about your thoughts about EMDR/ImRs?

Prompts What is helpful about therapy?

What is unhelpful about therapy?

Was there anything that you feel could been done differently? Maybe something added or left out?

6. Do you have any thoughts about who would benefit from this particular treatment?

Please explain why…

7. How do you think this treatment differs from other types of therapies for treating PTSD?
8. How helpful is this treatment for Complex PTSD?
9. Will you use this treatment in future?

**For therapists who use both treatments**

10. What are the differences / similarities between the two treatments?
11. What factors would influence your decision to use one treatment over the other?

**Difficulty/Barriers for Treatment**

**I want to ask you about any problems that you might have had during treatment**

12. Did you encounter any issues or challenges during treatment?

Were you able to overcome these difficulties?

How?

13. Have you experienced any difficulty or discomfort personally during or after therapy sessions?

Tell me more…

14. Were you hesitant to begin or continue treatment with a participant at any stage during the trial?

If yes, why?

15. Did any of your participants discontinue treatment before the end of the trial?

What reasons did they give?

What are your thoughts on this?

**Therapist Factors**

**The next questions I am going to ask a few questions related to therapist factors**

16. Have you discussed your experience of treatment with other therapists? What did you learn from this?
17. What similarities did you have in your experiences? Did you have any differences?
18. Do you have any advice for other therapists?

**Trauma-Focused Approach/Childhood Trauma-Related PTSD**

**The next questions are related to treatment of Complex PTSD. Firstly, I have some definitions outlining the different types of approaches to treating childhood trauma-related PTSD.**

19. There is a lot of debate in the research about the best/most appropriate way to treat PTSD related to childhood trauma. What is your understanding of this?
20. Do you have an opinion on how to best treat this disorder?

**Some people think that trauma-focused treatment is not appropriate for people who have experienced childhood trauma.**

21. What is your opinion on this?
Prompts Do you agree with this?
Why/ why not?
22. What factors would influence your decision of whether to adopt a trauma-focused approach for treating Complex PTSD?

**Research Trial specific - Experience/Opinion**

**Now I want to ask you questions specifically related to the research trial**

23. What was your experience of participating as a therapist in the research trial?
Have you ever been involved in a research project before?
24. What made you agree to participate in this trial?

**The format of the research trial was 2, 90-minute sessions, twice a week for approximately 6 weeks.**

25. What is your opinion on this format?
26. What about 2 treatment sessions per week?
27. Do you think that 12 sessions were appropriate? Maybe more or less? If so how many? Did you finish treatment early with anyone?
28. After you completed the 12 treatment sessions, there was an 8-week period without any treatment or follow up, what is your opinion on this?
29. Having participated in the study has your views, attitude or beliefs changed in anyway?
How?
30. Has your involvement in the research trial changed your practice working with childhood trauma?
31. Has this study influenced your view on 'the right time' to begin trauma-focused treatment?

**Any other comments**

**One of the aims of this study is to explore factors that influence treatment or the implementation of treatment to see how we can help improve things for the future.**

32. Keeping that in mind, what do you think are the 3 or 4 key things that you have learnt from your experiences that have influenced your practice?

**This brings us to the end of the interview.**

33. Do you have any other comments you would like to make?
